# Asynchronous Onset of Clinical Disease in BSE-Infected Macaques

**DOI:** 10.3201/eid1907.120438

**Published:** 2013-07

**Authors:** Judith Montag, Walter Schulz-Schaeffer, Annette Schrod, Gerhard Hunsmann, Dirk Motzkus

**Affiliations:** German Primate Center, Göttingen, Germany (J. Montag, A. Schrod, G. Hunsmann, D. Motzkus);; University of Göttingen, Göttingen (W. Schulz-Schaeffer)

**Keywords:** Prion disease transmission, bovine spongiform encephalopathy, BSE, Macaca fascicularis, prions, Creutzfeldt-Jakob disease

## Abstract

To estimate the effect of the variability of prion disease onset on primary bovine spongiform encephalopathy transmission to humans, we studied 6 cynomolgus macaques. The preclinical incubation period was significantly prolonged in 2 animals, implying that onset of variant Creutzfeldt-Jacob disease in humans could be more diverse than previously expected.

Prion diseases, such as bovine spongiform encephalopathy (BSE) in cattle, scrapie in sheep, and Creutzfeldt-Jakob disease (CJD) in humans, are fatal, transmissible, neurodegenerative disorders associated with the aggregation of an infectivity-associated isoform (PrP^Sc^) of the cellular prion protein (PrP) (*1*). Seventeen years ago, it became apparent that the BSE-infectious agent had entered the food chain and was identified as the causative agent for a new variant CJD (vCJD) (*2*). Since then, several risk assessment studies have investigated the number of expected vCJD cases in human populations (reviewed in [*3*]). Although thousands to millions of consumers of beef products were estimated to be affected, thus far only a few more than 200 vCJD cases have been observed worldwide.

This discrepancy was assumed to be attributable to the so-called species barrier, defined as the hindrance of an infectious agent to change its natural host. Upon crossing the species barrier, prion diseases often show a low attack rate in conjunction with a high variability in the preclinical incubation time. Thus, the consumption of BSE-contaminated products may have led either to a restricted infection or to a prolonged asymptomatic phase in some exposed persons. Therefore, concerns have been raised that asymptomatic carriers of vCJD might exist, posing a risk for unintentional human-to-human transmission.

First indications that transmission of BSE to primates may lead to variances in the preclinical incubation times were obtained by inoculating cynomolgus macaques with cattle-derived BSE material (*4*–*6*), even though in those studies not more than 3 animals were used. We have now used a group of 6 macaques that were infected with BSE at a comparable age and kept under identical and controlled experimental conditions.

## The Study

Six captive-bred female cynomolgus macaques (*Macaca fascicularis*, purchased from the Centre de Recherche en Primatologie, Mauritius) were inoculated intracerebrally with 1 dose of 50 mg brain homogenate (10% wt/vol) derived from 11 BSE-infected cattle. Animal experimentation was performed in accordance with section 8 of the German Animal Protection Law in compliance with Directive 86/609/EEC. Macaques were housed in a social group, and behavioral changes were assessed on a daily basis by experienced animal care takers.

After inoculation, all 6 macaques remained healthy and asymptomatic for >30 months (Table). At 931 days postinfection, 1 animal showed indications of slight coordination disorders. Within a few days, afferent ataxia developed, and when the animal was separated from the others animals, she apparently became tame. After 2 weeks, the animal showed severe dysmetria of the extremities without obvious myoclonia. Dementia was apparent but could not be diagnosed by objective measures. For ethical reasons, the animal was euthanized 17 days after disease onset. Within the next 14 weeks, 3 more animals became symptomatic. After appearance of neurologic symptoms (ataxia, tremors), the affected animals were occasionally separated from the group when symptoms became more severe or attacks from asymptomatic animals occurred. The disease course in these animals was comparable to that of the first animal, but the progression was slower (91–103 days).

**Table:** Incubation periods of cynomolgus macaques infected intracerebrally with 50 mg brain homogenate from bovine spongiform encephalopathy–infected cattle*

Animal	Haplotype at codon 129	First clinical signs, dpi	Duration of clinical phase, dpi
A1	M/M	931	17
A2	M/M	1,398	103
A3	M/M	946	91
A4	M/M	1,025	94
A5	M/M	1,340	143
A6	M/M	946	103

*dpi, days postinfection.

**Figure 1:**
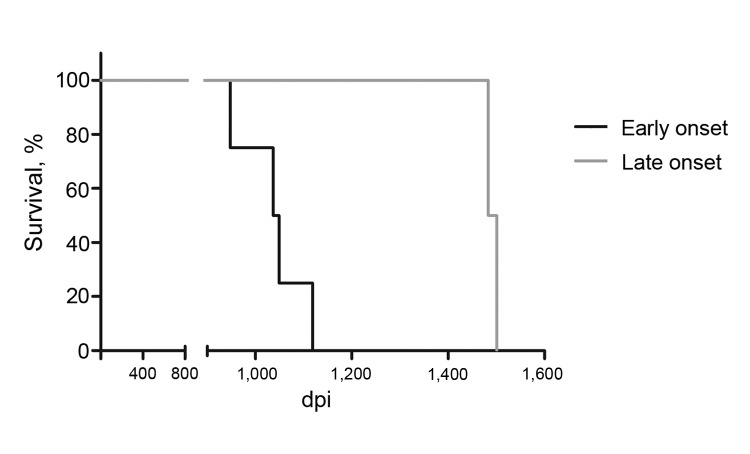
Survival of intracerebrally BSE-infected cynomolgus macaques. Six age- and sex-matched cynomolgus macaques were inoculated intracerebrally with 50 mg brain homogenate (10% in sucrose) derived from 11 BSE-infected cattle. Macaques were euthanized at severe signs of neurodegenerative disease. The animals were grouped according to early (<1,200 dpi, n = 4) or late (>1,200 dpi, n = 2) onset of disease. The respective survival curves were compared by using a log-rank test (Mantel-Cox, p<0.05). BSE, bovine spongiform encephalopathy; dpi, days postinfection.

Two of the 6 animals remained asymptomatic for ≈1 additional year. Although daily monitoring was facilitated by the fact that only 2 macaques remained and that the caretakers were more experienced to recognize minor changes in behavior, symptoms were first detected 1,340 and 1,398 days postinfection, respectively. Clinical signs were similar to those observed in the previous 4 animals. The symptomatic periods before euthanasia for these macaques lasted 103 and 143 days, respectively (Table). Direct comparison revealed that the difference between the short (931–1,025 days) and the long (1,340–1,398 days) preclinical incubation time was statistically significant (Figure 1, log-rank [Mantel-Cox] test, p<0.05).

Test results of brain samples from all animals were positive for macaque-adapted BSE by Western blot analysis. In brief, brain tissue from each animal was homogenized and subjected to proteinase K (PK) treatment for 1 h at 37°C. Samples were separated on acrylamide gels and transferred to nitrocellulose membranes. Macaque-adapted BSE (PrP^Sc^) was detected by using the monoclonal anti-PrP antibody 11C6. PK-resistant PrP was detected in all 6 macaques, confirming that BSE was transmitted to the animals.

**Figure 2:**
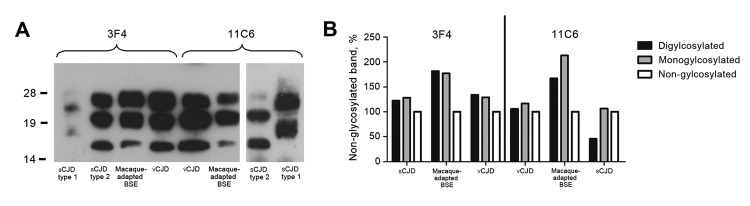
PrP^Sc^ profile of macaque-adapted BSE in comparison to human CJD. Brain homogenates from human sCJD type 1, sCJD type 2, vCJD, and BSE-infected macaques were subjected to PK treatment, separated on 12% sodium dodecyl sulfate–polyacrylamide gel electrophoresis, and blotted onto nitrocellulose membranes. A) PrP^Sc^ for human and macaque brain was detected with the widely used monoclonal antibody 3F4 or with 11C6. B) Glycoform ratio of sCJD type 2, vCJD, and macaque-adapted BSE. The relative signal intensities of the diglycosylated, monoglycosylated, and nongylcosylated isoforms were determined densitometrically and normalized to the band of the nongylcosylated isoform. PrP^Sc^, prion protein isoform; BSE, bovine spongiform encephalopathy; CJD, Creutzfeldt-Jakob disease; sCJD, sporadic CJD; vCJD, variant CJD; PK, proteinase K.

The individual glycopattern and band migration of macaque-adapted PrP^Sc^ was compared with human sporadic CJD (sCJD) type 1, sCJD type 2, and vCJD. PK-resistant PrP from BSE-infected macaques co-migrated with type 2 sCJD and was clearly distinguishable from type 1 sCJD (Figure 2). The glycosylation pattern of macaque-adapted BSE was comparable with vCJD (*6*,*7*), which is characterized by an overrepresentation of diglycosylated PrP^Sc^ (*8*,*9*). Using 11C6 antibody (*10*), we detected a slightly decreased signal of the diglycosylated PrP^Sc^ isoform for sCJD, vCJD, and macaque-adapted BSE. We assume that this effect is related to a reduced affinity of the diglycosylated isoform to 11C6 that otherwise shows high sensitivity to macaque-adapted PrP^Sc^. Nevertheless, direct comparison showed a higher amount of the diglysosylated PrP^Sc^ isoform in vCJD and macaque-adapted BSE than sCJD, which was also shown with a different monoclonal antibody, 3F4. This finding confirms that BSE transmission to macaques is comparable with, and can be used as a model for, human vCJD infection.

## Conclusions

Several susceptibility studies using nonhuman primates as a model for human prion diseases hint to heterogeneity of the preclinical incubation period upon crossing the species barrier (*5*,*11*,*12*). However, because of the low number of no more than 3 animals, this variability was not always evident (*4*). Therefore, there was an urgent need to determine whether the transmission of BSE to humans is likely to lead to a similar diversity.

Our study using 6 cynomolgus macaques shows that the transmission of BSE to primates led to a significantly prolonged asymptomatic phase in 2 animals. Disease onset is influenced by several factors (*13*). Our study design enabled us to exclude that the route of transmission influenced the disease progression because the infectious agent was injected into the same brain region of each animal. Also, a limited infectious dose cannot be responsible, as shown by the attack rate of 100%. In addition, endogenous factors, such as age, the MM genotype at codon 129 (Table), and housing conditions, were comparable for all macaques.

Thus, we conclude that the variable asymptomatic phase is most likely influenced by the infectious agent (*14*) or the genomic diversity of the macaques (*13*). The animals in our study were not inbred. Therefore, differences in the genomic background may have influenced the time of disease onset. In contrast, the PrP^Sc^ migration patterns of the animals give no indications for different types or strains that evolved from the mixed BSE inoculum. However, further studies will have to verify this.

Nevertheless, during the BSE epidemics, the human population with its natural genomic diversity was also exposed to a nonhomogenous prion source. Therefore, our study closely mimics the human situation. Our results imply that a prolonged asymptomatic phase can be expected for vCJD. In light of the transmissibility of vCJD through blood transfusions (*15*), our findings emphasize the need for continued attention to the risks of secondary human-to-human transmission.
